# Microlearning mApp raises health competence: hybrid service design

**DOI:** 10.1007/s12553-015-0095-1

**Published:** 2015-02-24

**Authors:** Luuk P. A. Simons, Florian Foerster, Peter A. Bruck, Luvai Motiwalla, Catholijn M. Jonker

**Affiliations:** 1 Delft University of Technology, Delft, The Netherlands; 2 Georgia Institute of Technology, Atlanta, GA USA; 3 Research Studios Austria Forschungsgesellschaft mbH, Wien, Austria; 4 Operations & Info Systems Department, University of Massachusetts Lowell, Lowell, MA USA

**Keywords:** Mobile health, mApp, Service design, Multi-channel services, Healthy lifestyle intervention, Self-management, Health competence

## Abstract

Work place health support interventions can help support our aging work force, with mApps offering cost-effectiveness opportunities. Previous research shows that health support apps should offer users enough newness and relevance each time they are used. Otherwise the ‘eHealth law of attrition’ applies: 90 % of users are lost prematurely. Our research study builds on this prior research with further investigation on whether a mobile health quiz provides added value for users within a hybrid service mix and whether it promotes long term health? We developed a hybrid health support intervention solution that uses a mix of electronic and physical support services for improving health behaviours, including a mobile micro-learning health quiz. This solution was evaluated in a multiple-case study at three work sites with 86 users. We find that both our mobile health quiz and the overall hybrid solution contributed to improvements in health readiness, −behaviour and -competence. Users indicated that the micro-learning health quiz courses provided new and relevant information. Relatively high utilization rates of the health quiz were observed. Participants indicated that health insights were given that directly influenced every day health perceptions, −choices, coping and goal achievement strategies, plus motivation and self-norms. This points to increased user health self-management competence. Moreover, even after 10 months they indicated to still have improved health awareness, −motivation and -behaviours (food, physical activity, mental recuperation). A design analysis was conducted regarding service mix efficacy; the mobile micro-learning health quiz helped fulfil a set of key requirements that exist for designing ICT-enabled lifestyle interventions, largely in the way it was anticipated.

## Introduction

Work place health support interventions are very relevant: to support our aging work force and to help reduce the private and social burdens of preventable disease. There are two design challenges there: increasingly more support is required, and this has to be delivered at lower costs. Hence, there is an urgency to develop automated eHealth solutions to improve user’s health.

Previous research highlighted the following requirements in the work context: solutions need to be efficient; they should limit their intrusion on the other work tasks [3]; they should offer enough newness and relevance [24] each time the mobile health application is used. Otherwise the eHealth law of attrition applies: 90 % of users are lost prematurely [7].

This eHealth law of attrition is an important design challenge. On the one hand, we know that many health improvement initiatives of people fail and that with extra support, success rates improve [1, 13]. On the other hand, automated eHealth support is often the economically preferred way (especially in the future, given our aging population). But if eHealth applications generally lose 90 % of their users within several usage instances [7], we are not adding enough value with eHealth.

In previous research [23] we have shown that various mobile health applications, even when they are popular mApps from the App stores, still face challenges in delivering added value in existing health coach relationships. In several of our real world user tests, the ‘eHealth law of attrition’ applied [7]. In other words, a large majority of users (>90 %) stopped using the mApps within 2 weeks and judged that they added little new information or value after a few initial usage events. Very similar patterns were found for a food choices app (CalorieTeller), physical activity (Runkeeper) and a stress management app (Pranayama). Two main design lesson conclusions were [24, 25]: First, part of the attractiveness of an mApp appears to be the ‘newness factor’: do I learn or experience something new? Second, it helps when the mApp is a core element of a larger service mix within an existing health coach relationship, see also [11].

Moreover, as part of qualitative user feedback, we found a continuous hunger from health coach participants for new health information. It was decided to create a health coach service mix, where the processes of health education would be supported with a mobile health education system in the form of a micro-learning Health Quiz on multiple health behaviour themes (food, physical activity, mental energy, long term sustainable health behaviours).

The high-level research questions we ask are: Can a mobile health-quiz provide added value within a hybrid (electronic and face to face) health coach service mix? Do mobile health-quiz app promote long term health behaviour, readiness (awareness, motivation, plans and actions) and health competence? To answer these questions we conducted a multiple-case study in three different work site environments (employers) and addressed the research question via four specific questions: First, does the mobile health quiz provide added value (usefulness, fun, positive triggers) with low barriers (ease of use, limited time burdens)? Second, can the mobile health quiz be integrated in effective health coach processes and overall service mix? Third, do the mobile health quiz and the overall service mix improve health readiness (awareness, motivation, plans and actions) and health competence (health perceptions, everyday choices, coping and goal achievement strategies, growth and health identity)? Fourth, do the mobile health quiz and the overall service mix improve health behaviours, short term and long term?

## Theory

For health coaching solutions to be effective, they need to help improve health readiness as indicated in the HAPA (Health Action Process Approach) model [16, 14] and i-change models [6]. Three important phases are distinguished. Barriers or motivators for change can reside in each of these phases, which are: awareness, motivation/intention, and practice (including planning, experiencing, coping, improving). And as an underlying theme self-efficacy is important in these models, namely: can we support people in developing skills to live more healthily and with tactics to deal with challenges?

In practice, around 90 % of daily perceptions and choices occur largely automatically, using a set of competences that Kahneman [12] describes as made by the ‘system 1’ fast decision making parts of our brain. This includes perceptions and choices related to healthy lifestyle. This regards choices like:

‘Did you come to work by bicycle this morning? What did you have for breakfast? Did you brush your teeth?’ and ‘How long did you take to pause and think about these choices?’

If you make healthy choices ‘on auto pilot’ they require little effort (or self-control or will power, [2]). Then they have a larger chance to become long term health patterns, especially if reinforced by self-evaluative perceptions of health identity, preferences and quality of life [12] and patterns of personal growth [18]. Collectively, these skills and patterns form a health competence set. Interestingly, the higher people’s health competence levels, the higher their happiness levels appear to be [10]. In summary, this health competence set consists of health perceptions, everyday choices, coping and goal achievement strategies, growth and health identity. Like any competence set, these are open to training and development [18], which is the aim of our health coach intervention.

**Fig. 1:**
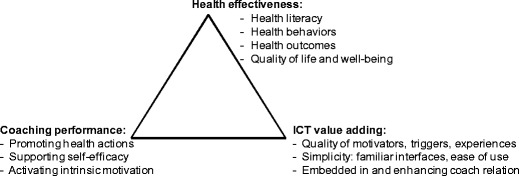
Basic requirements when designing ICT-supported healthy lifestyle interventions

If we look at the design challenge of persuasive technology [8, 9] for health, it was theorized and tested elsewhere that this challenge is not just located in the ICT design, but also in the design of the overall service scape, including health effects and coach relationship [25]. It should generate positive, mutually reinforcing service experiences across communication channels and activate long term health motivation and -behaviours, in order to deliver long term results. This is reflected in the following design evaluation framework for health improvement ICT solutions [25], see Fig. 1. It evaluates the impact of the ICT-enabled intervention on health effectiveness, coaching performance and ICT value adding.

To increase solution impact, a hybrid or multi-channel service mix is recommended [29, 28, 22–24], combining electronic and face-to-face interactions. For example, face to face ‘on site’ coaching had as benefits: a richer service experience with the coach, with other participants and with a health focused ‘service scape’; group support experiences (obtaining additional social support and co-creating service experiences together); learning from each other; health experiences in healthy food-, sports- and relaxation exercises. Disadvantages are: more (travel) time needed; less flexibility regarding when and where; and not everyone likes group sessions [5]. Electronic and (semi-)automated coaching has as benefits: more time-efficient; more flexibility in when and where to have contact; very explicit monitoring of your own progress online; having status reports including ‘next steps’ commitments always online. Disadvantages are: the sensory-, emotional- and group experiences are more limited. Also, the ‘service scape’ in which people are immersed is only virtual, not physical. In summary, often a hybrid service mix has most to offer.

In such a hybrid service mix, mobile micro-learning could potentially offer a number of advantages, namely, it uses a personal device that is available any time any place, it is efficient and can use idle time that is otherwise lost, and it is suited for just in time learning [3].

## Methods and materials

Regarding our design research approach, we follow the design cycle methodology provided by Vaishnavi & Kuechler W.[28]) which goes from problem awareness and solution suggestion to development, evaluation and conclusion. After reporting our multiple-case study results in section 4, we will discuss design lessons in section 5.

### Intervention: hybrid lifestyle intervention with mHealth App

The mHealth Health Quiz App was used within a hybrid (face-to-face and digital) service mix. The healthy lifestyle support service mix consisted of:

- Digital health behaviour surveys ‘BRAVO’ with automated personal feedback (on physical activity, smoking, alcohol, food and energy/recuperation), at 0, 1, 3 and 10 months.
- A 3-hour start up workshop (with about 30 participants)
- Personal action plan drawn up at the start workshop
- Asking participants to use weekly buddy contacts
- The mHealth Health Quiz App (available for Android, iOS, blackberry and laptops)
- Supporting health education materials via email
- A 2-hour repeat workshop after 1 month for answering questions and for (peer) education
- Twelve times a weekly health tip email to help maintain awareness and motivation

Our Health-Quiz App was based on the micro-learning principles and technology platform as outlined previously [3]. On this platform, learning occurs via micro learning cards, each containing a question, (mostly) multiple choice answer options, plus a brief explanation after each answering attempt. The learning cards are organized in a number of courses and participants can see how much they progressed within a course. The course content was designed with seven course modules of about 20 questions each. The first three courses educate participants on the ‘what’ or basic knowledge of healthy food-, exercise- and mental energy behaviours. The fourth course provides education and examples on what determines long term health behaviour success. Courses five through seven educate participants on the ‘how’ or daily tactics of healthy food-, exercise- and mental energy behaviours.

Within the course content design, questions are ranked in a certain order to address consecutive learning objectives. First, basic knowledge and awareness are increased, then motivation and plan making, next there is support for daily activities and coping strategies, plus seeking new self-norms and self-identity. Participants are free to switch between courses in order to support ‘just in time’ learning based on their needs. Our Health Quiz Apps were downloaded and activated for all users during the 3-hour start up workshop.

### Multiple-case study in three employer organizations

From Feb to Dec 2013, a multiple-case study was conducted with three employers to evaluate real world impacts of the healthy lifestyle intervention in Dutch work settings. The employers were: a Municipality (*n* = 30 participants), an Advocacy organization for Dutch senior citizens (*n* = 26 participants, half of them volunteer workers) and a Care Provider (*n* = 30 participants).

From the perspective of these three organizations, an important question was: does this lifestyle intervention generate health behaviour improvements in the short and longer term? This was assessed with a standardized Dutch online ‘BRAVO’ survey (on physical activity, smoking, alcohol, food and energy/recuperation), at 0, 1, 3 and 10 months. Moreover, at months 1, 3 and 10 several additional health behaviour and health readiness (according to the stages of awareness, willingness, plans and actions) questions were asked on top of the brief BRAVO question set.

Next, a design evaluation survey was added at month 1, and the findings from this survey were discussed in the 1-month group workshops at each organization, to obtain qualitative insights into the why and how of the survey data and user experiences. The design evaluation survey addressed: 1) an evaluation of health promotion value of the different elements in the service mix, including the Health Quiz App, 2) the micro-learning mApp usefulness and ease of use. Besides, logging was conducted of use and progression in the micro-learning mApp environment.

## Results

The overall patterns were rather similar across the three employer organizations regarding: adoption, evaluation and effects of the healthy lifestyle intervention. Hence, we first describe the generic outcomes. After that, we report cross-case differences.

At the start, there were 86 participants (*n* = 30/26/30 for Municipality, Advocacy and Care Provider respectively). The survey response rates were 74 % (*n* = 64) at 1 month, then 55 % (*n* = 47) at 3 months and 56 % at 10 months (*n* = 48 for the BRAVO questions, *n* = 47 for the rest).

### Generic outcomes across the three employer organizations

From the perspective of the three employer organizations, the key outcome is that health behaviours improved after using our Health-Quiz app, as measured with the standardized Dutch BRAVO survey (on physical activity, smoking, alcohol, food and energy/recuperation). As expected, the main improvement in health behaviour patterns occurs in the first month. After that, the new pattern is relatively stable, at least for the 10 months period we measured. This pattern was observed within each organization. Table 1 provides the behaviour distribution accumulated for the three organizations at the start and 10 months. Table 2 summarizes the number of worsened and improved scores at months 1 and 10, compared to the start. Green means: compliant with Dutch health norms, yellow is nearly compliant and red is the rest.

**Table 1:**
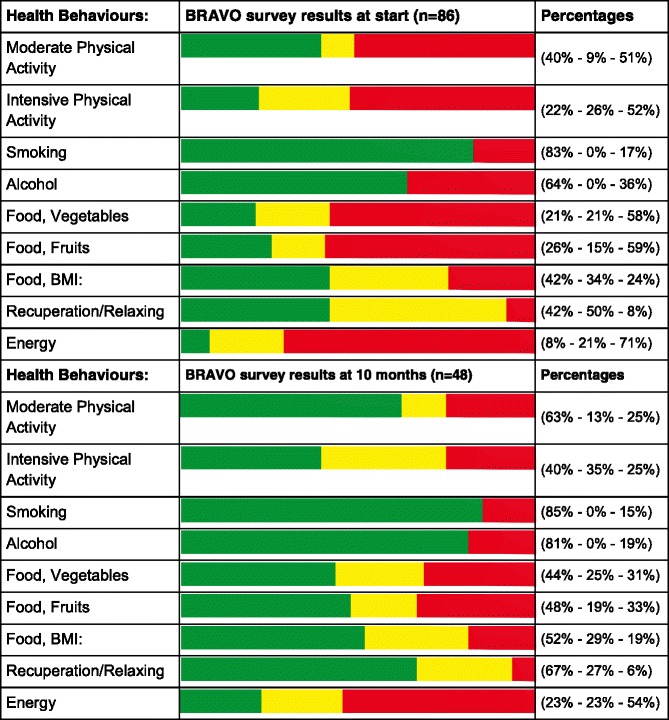
Health behaviours distribution at start and 10 months

Green = health norm compliant. Yellow = nearly compliant. Red = the rest

**Table 2:** Summary of worsened and improved behaviour scores at 1 and 10 months (irt start)

	Number of people, at 1 month (*n* = 64)		Number of people, at 10 months (*n* = 48)	
Health Behaviours:	Worsened:	Improved:	Worsened:	Improved:
Moderate Physical Activity	4	19	4	14
Intensive Physical Activity	4	21	4	18
Smoking	2	2	1	3
Alcohol	4	8	1	8
Food, Vegetables	2	23	3	16
Food, Fruits	5	32	1	18
Food, BMI:	2	4	1	5
Recuperation/Relaxing	5	16	3	14
Energy	5	17	5	14

Additional behaviour improvements reported in the surveys were (we list how many of the *n* = 47 respondents (strongly) agreed at 10 months): I am physically active more often during the day (72 %), I take a relax moment more often (47 %), I more structurally reduce my stress sources (51 %), I eat fewer sugars and refined carbohydrates (55 %), I eat more whole meal plant foods like nuts, mushrooms, olives etc. (81 %), I eat less red and processed meat (57 %), I eat fewer butter fats (60 %). Next, the health readiness indicators improved (awareness, intentions and plans), plus health effects in terms of improved physical or mental fitness. The scores at 1, 3 and 10 months were very similar. Table 3 provides the 10 months results (*n* = 47).

**Table 3:**
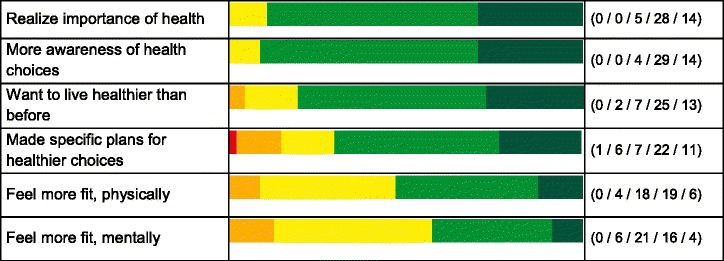
Health readiness and fitness improvements after 10 months, *n* = 47

Green = (highly) agree. Yellow = neutral. Red/orange = (highly) disagree

Next, a question was: how did the Health Quiz App and other service mix elements contribute to the results above? This question was part of the 1-month evaluation. In the survey data as well as the workshops it was observed that not everybody had preferences for the same service elements. Still, most service elements were judged to be helpful for improving health behaviours by a significant part of the users, see Table 4. The lowest helpfulness scores as percentage ‘(strongly) agree’, *n* = 64, were given for: doing it as a group (20 %), having a buddy (25 %) and the weekly health tip mail (36 %). The highest helpfulness scores as percentage ‘(strongly) agree’, *n* = 64, were given for: having a second workshop at 1 month (52 %), feeling better (53 %), the Health Quiz mApp (53 %), making my own health activity plan (66 %), knowing what my own influence is (75 %) and the start workshop (77 %).

**Table 4:**
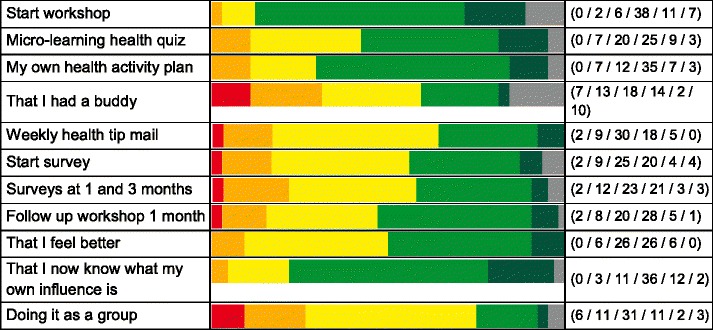
Service elements that stimulated healthier behaviours (*n* = 64, at 1 month)

Green = (highly) agree. Yellow = neutral. Red/orange = (highly) disagree. Grey = not applicable

A next set of evaluation questions at 1 month regarded usefulness, added value and ease of use of the micro-learning Health Quiz mApp. The majority of respondents indicated that it was efficient, useful, fun, that they would have learned less without it and that they now made healthier choices thanks to the micro-learning Health Quiz mApp. Likewise, the majority also indicated that it fulfilled a desire to learn more after the start workshop, that it was low effort, that the (smaller) mobile screens were no hindrance, that most learning cards were relevant, that things were learned that were directly applicable and that the courses provided regular stimuli to make healthy choices. These findings were confirmed in the feedback during the 1-month workshops.

Moreover, the qualitative findings were corroborated via the micro-learning logging data, in the sense that the majority of participants who started the micro-learning Health Quiz mApp also completed all 7 available courses. In contrast with the eHealth law of attrition [7], completion rates were relatively high. On an intention to treat basis, 66 % of the courses were completed that were made available for the *n* = 86 starting participants.

### Cross-case differences

There were a few case-specific characteristics that had an impact on adoption patterns and compliance and response rates. However, the overall design evaluation findings were very similar across cases. In Table 5 we highlight the differences and below we list the differences in course completion rates, based on microlearning logging data.

**Table 5:** Cross-case differences

Employer case	Case-specific characteristics and findings
Municipality	- Relatively lower start- and completion rates, partly due to the fact that several participants were sent by their managers and did not participate on a voluntary basis. - For those who did participate, the relative improvements in eating vegetables and adding moderate intensity physical activity were larger than in the other cases.
Advocacy	- About half of the group were (senior) volunteers and the average age in this group was highest. Several sudden events happened in the lives of these participants, hampering workshop participation and course completion for several of them. -Initial health behaviour scores were highest in this group: for daily physical activity, for fruit and vegetables consumption. However, their energy was lower and stayed low. - About 50 % were retired: some were more busy than ever. Others reported that the topics of work related stress and energy were less relevant for them.
Care Provider	- The regional director was a strong health advocate, participant and initiator in this group. Start- and compliance rates were highest is this group. - This group had the lowest start fruit consumption, but the highest final score. - The improvements were largest in energy and recuperation behaviours in this group, as well as the reported gains in mental fitness (at 1, 3 and 10 months).

In the Municipality case (*n* = 30), 14 participants completed all seven courses, five completed several courses and 11 completed none (of which 7 participants never started the micro-training). The total completion rate was 53 % of all available course material. In the Advocacy case (*n* = 26), 18 participants completed all seven courses, and eight completed none (of which five never started the micro-training). Their total completion rate was 69 % of all available course material. In the Care Provider case (*n* = 30), 21 participants completed all seven courses, four completed several courses and five completed none (all of them did start the micro-training and completed multiple questions, mostly sampling them across multiple courses). The total completion rate was 75 % of all available course material.

## Discussion and conclusion

In conclusion, the short answer to all four research (sub-)questions in this paper was yes, and we will discuss the answers to the research questions in relation to the evaluation framework of Fig. 1. First, the mobile health quiz provides added value (usefulness, fun, positive triggers) with low barriers (ease of use, limited time burdens). Second, according to the participants the mobile health quiz is well integrated in the overall service mix. Hence, the ‘ICT effectiveness’ factor of Fig. 1 appears to be sufficiently addressed. The mobile health quiz also increases the ‘coaching effectiveness’ factor of Fig. 1 by providing regular triggers for health awareness, coping strategies and useful health behaviours. In answer to the third research sub-question, health readiness (awareness, motivation, plans and actions) and competence (health perceptions, everyday choices, coping and goal achievement, growth and development) are improved. Fourth, the various health behaviours are improved as measured with the Dutch BRAVO survey (on physical activity, smoking, alcohol, food and energy/recuperation).

The answers to the third and fourth research sub-questions demonstrate the contribution to the ‘health effectiveness’ factor of Fig. 1, both of the overall hybrid service mix and of the mobile micro-learning health quiz within that mix. Besides explicit participant feedback, we have the indications from the course completion rates of the mobile health quiz, which were 66 %: well above the Eysenbach [7] ‘law’. Given the fact that these courses were not mandatory for these participants, but only additional support for health self-management, and given the fact that each course easily takes 20 min to complete (in a context of time scarcity, [3]) we regard course completion rates as a sensible proxy for perceived usefulness.

Health behaviours not only improved at 1 and 3 months, but also 10 months after the start. The latter finding is relatively special, in the sense that most healthy lifestyle interventions, whether at work sites [29] or not [17], tend to generate only short term results (3 or 6 months), after which people generally fall back into their old patterns.

These long term effects might have been promoted by a positive peer support effect from other group members [15] or management (see ‘Care Provider’ case in Table 5), even though this was not mentioned as a strong factor by most participants: see last item of Table 4.

### Theory

Our *contributions to theory* are threefold. Through this empirical test of our proof of concept with employer organizations we see a tentative confirmation of the three design research propositions on which we have built our design research. First, a mobile micro-learning health quiz appears useful for fulfilling the key design requirements from Fig. 1 when designing ICT-supported healthy lifestyle interventions: health-, coaching- and ICT effectiveness.

Second, as contributions on these design requirements increased, we indeed empirically observed improved health readiness, behaviours and competence. Thus, our second proposition is tentatively confirmed that the design requirements from Fig. 1 may contribute to ICT-enabled health intervention success.

**Fig. 2:**
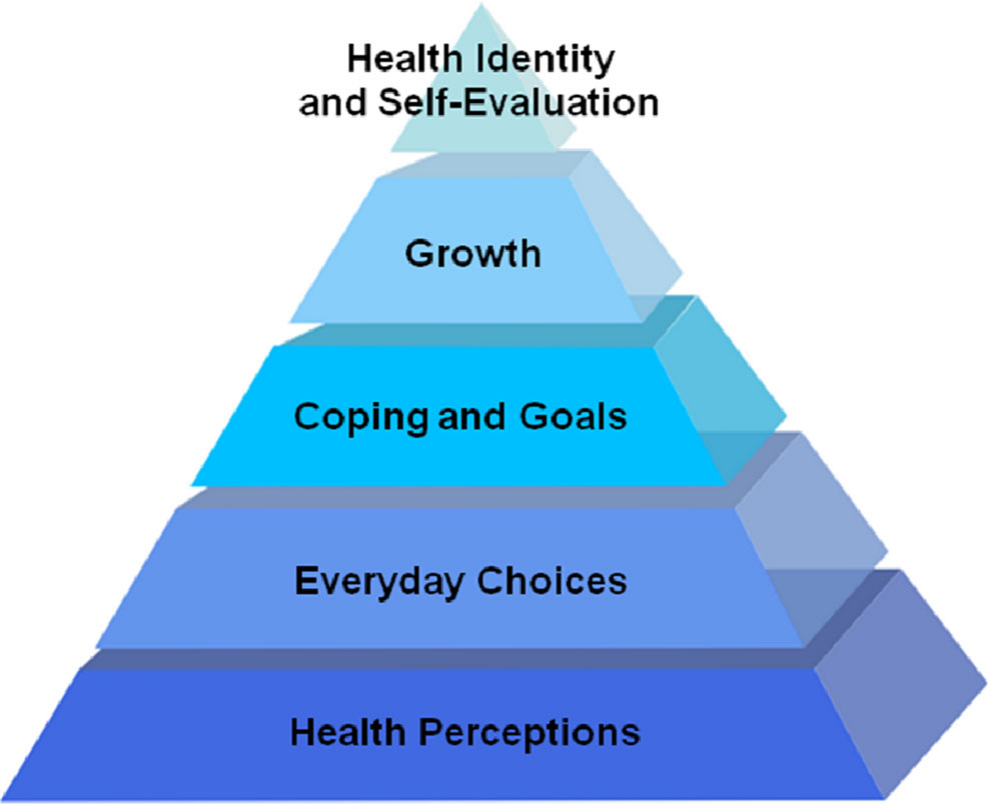
Health competence pyramid for long term health

Third, long term health behaviours appear to benefit from a grounding in health competences (health perceptions, everyday choices, coping and goal achievement, growth, health identity and self-evaluation), see Fig. 2. This is largely a qualitative observation, based on participant feedback and sensitized by the work of Kahneman [12] and Seligman [18] on daily routines, decisions, growth, and the way happiness and life satisfaction are grounded in identity and self-evaluation, see also the theory section. Long term health impacts seem to lie not only in health readiness (awareness, intentions, plans, actions – which have a relatively operational focus and are aimed at next week rather than next year, see the HAPA and i-change models from theory). Rather, longer term health competences can be trained and developed: from health perceptions, via everyday choices, strategies for coping and goal achievement, growth, to health identity and self-norms. This is also what several study participants indicated: their health views had changes, as well as their preferences, choices, health goals, self-management and -evaluation. They indicated this is what helped them deal with the changing dynamics of life and health in the longer run. We have planned additional research in order to more rigorously measure the direct contributions of eCoaching to health competence development.

### Limitations and practical implications

Regarding *practical implications*, the relevant design question is what made these results come about and how can we improve even further? After discussing study limitations we conduct a design evaluation, using the framework from our theory section.

This study has several limitations. First, it is largely qualitative, evaluating effects across three case organizations. In the survey, users mostly agree on issues of micro-learning perceived usefulness and perceived ease of use: both antecedents of the TAM model [4]. Statistical techniques like explorative regression analysis were not possible due to small sample size and low variance. Second, our survey results are likely subject to self-selection biases: our survey respondents were self-selected consisting of largely of users who completed most or all of the micro-learning courses. They are the ones most likely to be biased positively. Third, the context is different in each case organization and in our study design we cannot control for confounding factors. On the other hand, the strong evaluation- and effect agreement between participants across organizations does hint at the robustness and cross-case validity of the findings.

Table 6 summarizes results from our study and discusses methods for improvement for our design. Regarding factor 1 of Fig. 1, health effectiveness, more objective improvement measures could be advantageous. Especially for self-management of people with health issues (e.g., diabetes-2, irregular high blood pressure or heart arrhythmia, or impaired renal function) 24 × 7 monitoring of effects of lifestyle improvement can be very beneficial. For the second factor, coaching effectiveness, there is a challenge of automatically integrating context- and health information. For example, if we notice that someone has been sitting most of the day, when will reminders/triggers be appreciated to get up and move about, and when not? For example, if I’m very busy with finishing a report or having urgent meetings, it can be a conscious and preferred strategy to continue a sedentary work activity for the time being. Reminders and triggers can also become a nuisance. But at other times, when falling in my coach potato trap in the evening, I may very well appreciate more persistent triggers to go play sports with a buddy. Finally, ICT value adding (factor 3) could be improved via at least two routes. First, improving automated logging of health (behaviour) data and integrating this into coach processes. Second, designing more intelligent, interactive coach processes, which incorporate user preferences and plans, contextual/situational priorities and health data consequences.

**Table 6:** Design evaluation (authors’ opinions, 5-point scale from - - to ++)

Health Effectiveness	Coaching Performance	ICT Value Adding
++ Health Literacy: Impacts from Health Quiz, workshops and education materials. + Health behaviours: BRAVO survey indicates improvements. +/− Health outcomes: Feeling more fit is a positive outcome, but more objective measures not used. + Quality of Life: Feeling better, mentally and physically more fit.	+/− Promoting health actions: Many health tips are provided. Impact depends on execution of plans. ++ Supporting self-efficacy: Users indicate a strong contribution from ‘know what my own influence is’ ++ Activating intrinsic motivation: A strongly activated desire to improve, plus rewards via feeling better.	+ Motivators, triggers, experiences: health quiz, mail tips and surveys provided triggers, (fun) experiences plus hope and improvement opportunities. +/− Simplicity: Installation and first use were burdening for some. After that, usage was simple, low effort. +/− Fit with coach processes: Users felt synergies with the workshops, education materials, personal action plans and answering individual questions.
Potential improvement: Using more objective health outcomes, possibly future 24 × 7 health tracking.	Potential improvement: Context aware and personalized coaching.	Potential improvement: Coach processes could be automated more (e.g., goals/means support).

In summary, given the relatively static content of the micro-learning health quiz, it served its health support goals well, thanks to the other service mix elements and the overall service concept. eCoach improvement opportunities for the future abound, of which we identified several.
